# Why Do Goitered Gazelles Cross the Path of an Approaching Moving Object Instead of Fleeing?

**DOI:** 10.3390/ani16203323

**Published:** 2026-10-21

**Authors:** David Blank

**Affiliations:** Research Center for Ecology and Environment of Central Asia, Bishkek 720040, Kyrgyzstan; blankdavidalexander58@gmail.com

**Keywords:** goitered gazelle, road-crossing behavior, approaching objects, flight response, approach category

## Abstract

Animal–vehicle collisions are an important conservation concern. Although many studies examine road mortality and wildlife crossings, less is known about ungulates that run across the path of an approaching vehicle rather than simply fleeing. This study describes this behavior in goitered gazelles (*Gazella subgutturosa*) and examines its association with four recorded factors: approach category, group size, group composition, and month. In a forward stepwise logistic regression, approach category was associated with crossing behavior (Wald = 22.784, df = 2, *p* < 0.001). The odds of crossing were higher in the high-category group than in the low-category group (odds ratio = 2.36). However, the categories also differed in object type (cars, motor scooters, and bicycles/pedestrians), so the independent effect of speed cannot be separated from the effect of object type. Group size, group composition, and month did not meet the model’s entry criteria. These observational results identify an association, but do not determine whether path-crossing is a uniquely anti-predator response, a reaction to limited assessment time, or another form of behavior.

## Simple Summary

This study describes an unusual road-related behavior in goitered gazelles (*Gazella subgutturosa*): under some circumstances, animals run across the path of an approaching moving object rather than simply moving away. Similar behavior has been reported in several ungulate species in open habitats. Based on observations made in southeastern Kazakhstan, this paper describes the behavior and examines whether its occurrence was associated with the category of approaching object, group size, group composition, and month. The observations show an association between the recorded approach category and the gazelles’ response, but they do not establish the behavior’s function or evolutionary origin.

## 1. Introduction

Animal–vehicle collisions are a significant problem, particularly on heavily trafficked roads [1]. Road infrastructure, traffic volume, and vehicle speed contribute to wildlife mortality [1,2,3]. Although road mortality has received substantial attention, the behavioral responses of wild animals to approaching vehicles remain less well understood [1]. Factors associated with collision risk include landscape characteristics, traffic, and seasonal behavior [4]. Some ungulates avoid roads, including North American elk (*Cervus canadensis*) and reindeer/caribou (*Rangifer tarandus*), which may avoid even lightly used roads [5,6,7,8,9]. Reindeer/caribou may nevertheless have to cross roads during seasonal migrations, when their routes follow predictable paths [10,11,12]. In northern Alaska, pregnant female caribou have been reported to avoid roads associated with industrial infrastructure more than non-parturient females and males [13].

Animals may become habituated to vehicles travelling along predictable routes, and vehicles on roads may be perceived as less threatening than unexpected off-road approaches [1,14]. Such responses can increase opportunities for animal–vehicle encounters. Ungulates nevertheless respond to approaching objects in ways that vary with the approach, the surrounding habitat, and the animal’s state. For example, Stankowich and Coss (2006) reported that the flight distance of Columbian black-tailed deer (*Odocoileus hemionus columbianus*) increased with faster and more direct human approaches [15]. A faster approach may reduce the time available for threat assessment, although this mechanism cannot be assumed to explain every response to vehicles.

A less commonly described response occurs when an ungulate runs alongside an approaching moving object and crosses its path before it passes. Such behavior has been reported in roe deer (*Capreolus capreolus*), American elk, reindeer, saiga antelopes (*Saiga tatarica*), and Mongolian gazelles (*Procapra gutturosa*) [1,16,17]. It has also been observed in goitered gazelles. The sequence of movements can appear directed—for example, an animal may run parallel to a moving object before crossing its trajectory—but the observations alone do not establish the motivation or function of the behavior. Table 1 describes the behavioral patterns recorded in this study.

Several factors could be associated with whether goitered gazelles cross the path of an approaching object or move away. Previous research on reindeer/caribou has reported differences in flight responses by reproductive state and sex [13], while a review by Lima et al. (2015) discusses the role of approach speed in ungulate responses [1]. Group members may also respond collectively when one or more animals begin to move. I therefore examined four recorded factors: approach category, group size, group composition, and month. These were treated as potential correlates of the observed response, not as established causes.

The study tested four expectations. First, crossing frequency might differ among groups classified by sex and age composition. Second, crossing frequency might vary by month, given seasonal changes in reproductive state and behavior. Third, the category of approaching object might be associated with crossing frequency. Fourth, larger groups might cross more frequently than smaller groups. These expectations were evaluated using the observations available; unrecorded factors could also have influenced the responses.

## 2. Materials and Methods

### 2.1. Study Area

I studied goitered gazelles responding to moving objects in Ili Hollow, southeastern Kazakhstan, from 1981 to 1987. The study was conducted in the Kapchagai conservation area (Taldy-Kurgan Oblast), where hunting and tourism were prohibited. During this period, the gazelle population increased from approximately 1500 to 5000 individuals [18]. The area is a gravel desert plain sloping from the foothills toward the Ili River. Dry riverbeds, karstic depressions, and hills create a varied landscape.

The climate is highly continental and arid, with summer temperatures reaching approximately 40 °C and winter temperatures falling to about −30 °C. Strong winds occur particularly in winter and early spring, and snow cover is uneven. The open plain supports sparse desert and dwarf shrubs, generally 20–30 cm high, while denser vegetation occurs near springs and along the Ili River [19,20]. Gazelles sometimes enter these thickets but, according to the author’s observations, usually leave them when alarmed [21]. The present study concerns observed responses to moving objects and does not experimentally test the function of these responses.

Vehicle tracks and dirt or gravel roads cross the valley in several directions. Traffic was limited during the study, with approximately one or two ranger vehicles per day and occasional tourist groups. Consequently, the observations may not represent gazelle responses under heavy traffic. Shepherd camps were present along the protected area’s boundaries during summer, and shepherds travelled mainly on horseback. Hunting and tourism were prohibited within the area, although occasional poaching was reported at its periphery [22].

Wolves (*Canis lupus*) were common in the study area and were considered the main predator of goitered gazelles. Red foxes (*Vulpes vulpes*) were also present and, according to field observations, posed a risk mainly to newborn gazelles. Asiatic wild asses (*Equus hemionus*) occurred on the plains, while Siberian ibex (*Capra sibirica*) and argali (*Ovis ammon*) inhabited the surrounding mountains and occasionally encountered gazelles near the plain–mountain boundary [21].

### 2.2. Methods

I used focal-animal observation and all-occurrence recording methods [23]. Behaviors were recorded in their order of occurrence using binoculars (8×) and a spotting scope (30–60×). Observations were conducted in different parts of the study area, and routes and travel directions were varied to reduce the likelihood of recording the same groups repeatedly. Each encounter with an individual gazelle or a group was recorded as a separate observation. Because individuals and groups could not be identified reliably, repeated observations cannot be ruled out; therefore, complete independence among encounters cannot be guaranteed.

I recorded the approximate distance between gazelle groups and the road. Crossing behavior was observed in groups encountered within approximately 100 m of the road; groups farther away generally remained still or moved away. A crossing event was recorded when a gazelle or group crossed the trajectory of an approaching object. The approaching-object categories were defined using estimated speed and object type: cars travelling faster than 35 km/h (high category), motor scooters travelling at 15–35 km/h (medium category), and bicycles or pedestrians moving below 15 km/h (low category). Because each category was associated with a different type of object, the effects of speed and object type cannot be separated in this dataset. Speeds were estimated in the field rather than measured instrumentally. Five encounters with mounted shepherds were excluded because the gazelles fled in all five cases. Group size and, where identifiable, group composition (female groups, male groups, or mixed-sex groups) were recorded. The unit of analysis was the group/encounter, not each individual within a group; a group showing a common response was counted as one observation.

### 2.3. Study Design and Sampling Method

Observations were collected from June 1981 to September 1987 (66 months), mainly under a predefined protocol, with some additional observations recorded opportunistically during travel within the study area. Car and motor-scooter observations were generally conducted once per month; bicycle and pedestrian surveys were conducted 15–20 times per month. Sessions lasted approximately 3 h, for a total of about 450 h across all types of moving objects. Vehicle sessions covered approximately 75 km, and bicycle/pedestrian sessions approximately 10 km. Surveys were conducted for 10 months per year, excluding January and February because snow restricted gazelle movements. The recorded encounters comprised 1750 flight responses (79.3%), 293 crossing responses (13.3%), and approximately 164 cases in which gazelles stopped and watched the moving object (7.4%), for 2207 encounters in total. The binary comparison of crossing versus fleeing does not include the watching response. The original manuscript also reports 30 cases excluded because of low-frequency categories.

### 2.4. Statistical Analysis

I used forward stepwise logistic regression to examine associations between the recorded factors and the observed response. The candidate predictors were approach category, group size, group composition (sex/age category), and month. For the binary response, crossing events were compared with flight responses; encounters in which gazelles stopped and watched were not part of this binary comparison. Because object type and estimated speed were linked in the sampling design, the approach-category coefficient should not be interpreted as an isolated causal effect of speed. Analyses were conducted in SPSS version 23.0, using *p* < 0.05 as the significance threshold. The exact *n* used in the final model was 2013, from which 1750 ran away and 263 crossed the road, excluding 30 cases of low-frequency categories. Group-composition analyses used only encounters with known composition.

The dependent variable was coded as a group/encounter response: crossing the trajectory of the approaching object versus fleeing from it. Encounters in which gazelles stopped and watched were excluded from this binary comparison. The group/encounter, rather than each animal, was the unit of analysis.

## 3. Results

Across 2207 recorded encounters, gazelles fled from the approaching object in 1750 cases (79.3%), crossed its trajectory in 293 cases (13.3%), and stopped to watch in approximately 164 cases (7.4%). The 293 crossing events involved cars in the high category (*n* = 120), motor scooters in the medium category (*n* = 138), and bicycles or pedestrians in the low category (*n* = 35). Double-crossing—crossing the trajectory and then crossing back in the opposite direction—occurred in 2.8% of crossing events (approximately 8 cases). After crossing, gazelles stopped to watch the object in 3.8% of crossing events (11 cases). Stotting was recorded in 2.7% (8 cases), and semicircular movements in front of slower-moving objects in 0.8% (2 cases) of crossing events (totaling 30 cases). These less frequent behaviors are descriptive observations; their definitions and counts should be interpreted cautiously because the original field protocol did not provide a detailed operational definition for each category. No vehicle-related mortality associated with this behavior was recorded during the observation period.

The forward stepwise logistic regression (Table 2) identified the three-level approach category as the only predictor that met the model’s entry criterion (Wald = 22.784, df = 2, *p* < 0.001). Group size (*p* = 0.619), group composition (*p* = 0.506), and month (*p* = 0.778) did not meet the entry criterion. In the fitted model, the high category (cars, estimated at >35 km/h) had higher odds of crossing than the low category (bicycles/pedestrians; odds ratio = 2.361; B = 0.859, SE = 0.227, Wald = 14.293, *p* < 0.001). The medium category (motor scooters, estimated at 15–35 km/h) did not differ significantly from the low category (B = 0.101, SE = 0.242, Wald = 0.175, *p* = 0.676). Because object type was confounded with speed category, these results show an association with the combined category and do not establish an independent effect of speed.

## 4. Discussion

In this dataset, group composition and month did not meet the entry criteria for the regression model. This result should not be interpreted as proof that sex, age, or reproductive state has no effect on crossing behavior, particularly because group composition was not known for every encounter and the sample size for each category may have differed. The available observations did not show a statistically detectable association for these predictors under the model used.

Group size also did not meet the entry criterion in the fitted model. This does not demonstrate that group size is biologically irrelevant; it indicates only that the analysis did not detect an association after considering the variables included in the model. Collective movement may occur when several groups join or follow one another, as described in other ungulates and in human road-crossing behavior [24]. Blackwell et al. [25] similarly reported that white-tailed deer crossed ahead of approaching vehicles in some encounters without detecting an association with group size or several other measured variables.

The approach category was associated with crossing behavior in the fitted model (Wald = 22.784, df = 2, *p* < 0.001). Crossing was more likely in the high category than in the low category, but the categories also differed in object type: cars were in the high category, motor scooters in the medium category, and bicycles/pedestrians in the low category. Consequently, the present analysis cannot distinguish a response to speed from a response to vehicle type, noise, approach characteristics, or other correlated features. The association is compatible with several explanations, including altered threat assessment, but it is not direct evidence of an anti-predator mechanism.

My observations suggest that gazelles encountered within approximately 100 m of the road sometimes reacted to moving objects, whereas groups farther away often remained still or moved away. This distance was an observational estimate, not a threshold tested with a controlled design. A comparable response has been described in roe deer near railway lines [17], but differences in species and study conditions prevent direct equivalence.

The open, undulating terrain of the Ili Valley may have allowed gazelles to detect approaching objects from a distance. During the observations, groups within roughly 100 m sometimes initiated movement, whereas groups farther away were often unresponsive. Because visibility, exact distance to the object, and approach angle were not recorded systematically, these observations should be treated as descriptive rather than as evidence of a fixed detection threshold.

The observed behavior differs from many road-crossing events described as incidental movements during grazing or travel [25,26,27]. In some settings, collisions occur when animals do not detect or respond to vehicles in time, including at night or in dense vegetation [28,29,30,31,32]. The present observations describe a different pattern in which some gazelles crossed the trajectory of an approaching object; however, the study did not measure collision risk or directly compare this behavior with other crossing types.

In some encounters, gazelles ran parallel to a moving object and then crossed its trajectory, and other members of the group sometimes followed. These observations suggest a repeated sequence of movements, but they do not establish that the behavior is coordinated for a particular function. Other ungulates also show varied flight paths in response to approaching vehicles or predators [1,33]. Comparative evidence can provide possible explanations, but it cannot demonstrate the function of the behavior observed in this study.

Ungulates may use zigzagging, sharp turns, and stotting during predator encounters [34,35,36,37,38]. Such behaviors have been discussed as possible ways to evade or confuse predators, and path-crossing has been reported in saiga and Mongolian gazelles [16]. These comparisons make an anti-predator interpretation conceivable, but the present study did not observe predation events during the recorded vehicle encounters and did not test whether crossing reduces capture risk. The proposed mechanism should therefore remain a hypothesis rather than a conclusion.

The path-crossing behavior observed here may resemble movements used by ungulates during predator evasion, but the data do not show that it evolved for this purpose. Gazelles crossed in front of some fast-moving objects more often than in front of objects in the low category; however, the object categories differed as well as their estimated speeds. The absence of crossing in the five recorded encounters with mounted shepherds is descriptive and may reflect prior experience or other differences between mounted people and vehicles, but the observations are too few to establish the cause.

Open habitats may make it easier for gazelles to detect approaching objects, and comparable crossing or circling movements have been reported in saiga and Mongolian gazelles [16,39]. Similar group responses have been described in reindeer/caribou and roe deer near railway lines [1,17]. These comparisons indicate that ungulate responses to linear infrastructure can be varied, but they do not show that the behaviors share the same mechanism.

Several explanations could account for the association between approach category and crossing behavior. A faster approach might reduce the time available for assessment; alternatively, the object’s type, sound, trajectory, or predictability could affect the response [40]. The observations do not distinguish among these explanations. The possibility that some movements include a play-like or catch-up element is also speculative, even though locomotor play has been described in goitered gazelles [41]. No behavioral experiment or direct measure of the animals’ motivation was included in this study.

The study has several limitations. Traffic was light, and gazelle responses may differ under heavier traffic. Distance from the approaching object, approach angle, visibility, and terrain at each encounter were not recorded systematically and could not be included in the statistical model. In addition, because object type and estimated speed category were linked, the analysis cannot attribute the observed association to speed alone.

## 5. Conclusions

In conclusion, the study documents instances in which goitered gazelles crossed the trajectory of an approaching moving object rather than fleeing. The recorded approach category was associated with this response, but speed could not be separated from object type, and the available data do not establish the underlying cognitive, anti-predator, or evolutionary mechanism. Explanations involving predator confusion, shortened assessment time, or locomotor play should be treated as hypotheses for future study, not as demonstrated functions of the behavior.

## Figures and Tables

**Table 1:** Crossing-path behavior observed in goitered gazelles in response to moving objects.

Behavioral Category	Description
Fast-moving objects (cars and motor scooters)	When a car or motor scooter approached along the road, gazelles sometimes ran parallel to it, with their tails raised, at a distance of up to approximately 100 m from the road, apparently attempting to overtake it. During some prolonged chases (up to 2–3 min), individuals ran with their mouths open and tongues partly protruding. They often approached the road at an angle and crossed the object’s trajectory just before it reached them; in some observations, crossing occurred only a few metres ahead of the object. After crossing, gazelles usually moved away from the road, sometimes stopping to look back. On some occasions they crossed back in the opposite direction. These are descriptions of observed sequences; their function was not established.
Slow-moving objects (bicycles or pedestrians)	Gazelles sometimes ran a short distance toward the path of a bicycle or pedestrian and crossed its trajectory. In other observations, they crossed along an arc, leaving the moving object behind, or ran directly across its path. These behaviors were recorded descriptively and were not assigned a specific motivation.

**Table 2:** Logistic regression analysis of the effect of vehicle speed on the probability of goitered gazelles crossing the vehicle’s path.

	B	SE	Wald	df	*p*	Exp (B)
Step 1 Speed			22.784	2	>0.001	
Speed medium	0.101	0.242	0.175	1	0.676	1.106
Speed high	0.859	0.227	14.293	1	>0.001	2.361
Constant	−2.513	0.193	169.448	1	>0.001	0.081

Vehicle speed (overall test)—22.784|2| > 0.001.

## Data Availability

The raw data supporting the conclusions of this article will be made available by the author on request.

## References

[1] Lima S.L., Blackwell B.F., DeVault T.L., Fernández-Juricic E. (2015). Animal reactions to oncoming vehicles: A conceptual review. Biol. Rev..

[2] Seiler A., Helldin J.O., Davenport J., Davenport J.L. (2006). Mortality in wildlife due to transportation. The Ecology of Transportation: Managing Mobility for the Environment.

[3] Putman R., Apollonio M., Andersen R. (2011). Ungulate Management in Europe: Problems and Practices.

[4] Kim K., Woo D.G., Seo H., Park T., Song E.G., Choi T.Y. (2019). Korea road-kill observation system: The first case to integrate road-kill data in national scale by government. J. Environ. Sci..

[5] Forman R.T., Sperling D., Bissonette J.A., Clevenger A.P., Cutshall C.D., Dale V.H., Fahrig L., France R., Goldman C.R., Heanue K. (2003). Road Ecology: Science and Solutions.

[6] Jaeger J.A., Bowman J., Brennan J., Fahrig L., Bert D., Bouchard J., Charbonneau N., Frank K., Gruber B., Von Toschanowitz K.T. (2005). Predicting when animal populations are at risk from roads: An interactive model of road avoidance behavior. Ecol. Model..

[7] Fahrig L., Rytwinski T. (2009). Effects of roads on animal abundance: An empirical review and synthesis. Ecol. Soc..

[8] James A.R., Stuart-Smith A.K. (2000). Distribution of caribou and wolves in relation to linear corridors. J. Wildl. Manag..

[9] Rowland M.M., Wisdom M.J., Johnson B.K., Kie J.G. (2000). Elk distribution and modeling in relation to roads. J. Wildl. Manag..

[10] Miller F.L., Jonkel C.J., Tessier G.D. (1972). Group cohesion and leadership response by barren-ground caribou to man-made barriers. Arctic.

[11] Padilla E.S.R. (2010). Caribou Leadership: A Study of Traditional Knowledge, Animal Behavior, and Policy. Doctoral Dissertation.

[12] Wilson R.R., Parrett L.S., Joly K., Dau J.R. (2016). Effects of roads on individual caribou movements during migration. Biol. Conserv..

[13] Cameron R.D., Smith W.T., White R.G., Griffith B. (2005). Central Arctic caribou and petroleum development: Distributional, nutritional, and reproductive implications. Arctic.

[14] Stankowich T. (2008). Ungulate flight responses to human disturbance: A review and meta-analysis. Biol. Conserv..

[15] Stankowich T., Coss R.G. (2006). Effects of predator behavior and proximity on risk assessment by Columbian black-tailed deer. Behav. Ecol..

[16] Sludskiy A.A. (1962). Interrelation between predators and prey. Proceedings of the Institute of Zoology.

[17] Bhardwaj M., Olsson M., Håkansson E., Söderström P., Seiler A. (2022). Ungulates and trains–factors influencing flight responses and detectability. J. Environ. Manag..

[18] Blank D., Yang W., Xia C., Xu W. (2012). Grouping pattern of the Goitered gazelle Gazella subgutturosa (Cetartiodactyla: Bovidae) in Kazakhstan. Mammalia.

[19] Lomonovich M.I., Yakovenko Z.V. (1963). Climate. The Ili Valley and Its Natural Resources.

[20] Berezovikov N.N., Belyalov O.V. (2005). The nesting birds of the Altyn-Emel National Park. Selevinia.

[21] Blank D. (2025). The Behavioral Biology and Conservation of Goitered Gazelles (Gazella subgutturosa).

[22] Blank D.A., Kovshar A.F. (1990). Persian gazelle. Rare Animals of Desert Regions.

[23] Altmann J. (1974). Observational study of behavior: Sampling methods. Behaviour.

[24] Faria J.J., Krause S., Krause J. (2010). Collective behavior in road crossing pedestrians: The role of social information. Behav. Ecol..

[25] Blackwell B.F., Seamans T.W., DeVault T.L. (2014). White-tailed deer response to vehicle approach: Evidence of unclear and present danger. PLoS ONE.

[26] Carbaugh B., Vaughan J.P., Bellis E.D., Graves H.B. (1975). Distribution and activity of white-tailed deer along an interstate highway. J. Wildl. Manag..

[27] Riginos C., Graham M.W., Davis M.J., Johnson A.B., May A.B., Ryer K.W., Hall L.E. (2018). Wildlife warning reflectors and white canvas reduce deer–vehicle collisions and risky road-crossing behavior. Wildl. Soc. Bull..

[28] Bashore T.L., Tzilkowski W.M., Bellis E.D. (1985). Analysis of deer-vehicle collision sites in Pennsylvania. J. Wildl. Manag..

[29] Finder R.A., Roseberry J.L., Woolf A. (1999). Site and landscape conditions at white-tailed deer/vehicle collision locations in Illinois. Landsc. Urban Plan..

[30] Bellis E.D., Graves H.B. (1971). Deer mortality on a Pennsylvania interstate highway. J. Wildl. Manag..

[31] Allen R.E., McCullough D.R. (1976). Deer-car accidents in southern Michigan. J. Wildl. Manag..

[32] Meisingset E.L., Loe L.E., Brekkum Ø., Mysterud A. (2014). Targeting mitigation efforts: The role of speed limit and road edge clearance for deer–vehicle collisions. J. Wildl. Manag..

[33] Lee E., Croft D.B., Ramp D., Coulson G., Eldridge M. (2010). Flight response as a causative factor in kangaroo-vehicle collisions. Macropods: The Biology of Kangaroos, Wallabies, and Rat-Kangaroos.

[34] Caro T.M. (1994). Ungulate antipredator behaviour: Preliminary and comparative data from African bovids. Behaviour.

[35] FitzGibbon C.D. (1990). Mixed-species grouping in Thomson’s and Grant’s gazelles: The antipredator benefits. Anim. Behav..

[36] FitzGibbon C.D. (1993). Cheetahs and gazelles: A study of individual variation in antipredator behaviour and predation risk. Physiol. Ecol. Jpn..

[37] Walther F.R. (1969). Fight behavior and avoidance of predators in Thomson’s gazelle (Gazella thomsoni Guenther 1884). Behaviour.

[38] Landeau L., Terborgh J. (1986). Oddity and the “confusion effect” in predation. Anim. Behav..

[39] Sokolov V.E., Lushchekina A.A. (1997). Procapra gutturosa. Mamm. Species.

[40] Brown C.L., Hardy A.R., Barber J.R., Fristrup K.M., Crooks K.R., Angeloni L.M. (2012). The effect of human activities and their associated noise on ungulate behavior. PLoS ONE.

[41] Blank D., Yang W. (2012). Play behaviour in goitered gazelles, Gazella subgutturosa (artyodactyla: Bovidae) in Kazakhstan. Folia Zool..

